# Consequences of SARS-CoV-2 Infection in Pregnant Women and Their Infants: A Systematic Review

**DOI:** 10.7759/cureus.32787

**Published:** 2022-12-21

**Authors:** Mohammed A Aljohani, Fahad M Albalawi, Bader M Albalawi, Sameer S Alghamdi, Essam H Alghamdi, Ali A Almahl, Hassan A Alagoul, Ahmed M Alamori, Ahmed Y Mobarki, Ibrahim M Hadi, Mohammed A Asiri, Ibrahim M Dighriri

**Affiliations:** 1 Department of Laboratory and Blood Bank, Alwajh General Hospital, Tabuk, SAU; 2 Department of Laboratory, Laboratories and Blood Bank Administration, Taif, SAU; 3 Department of Laboratory, Dammam Regional Laboratory and Blood Bank, Dammam, SAU; 4 Department of Laboratory and Blood Bank, King Fahad Hospital, Jeddah, SAU; 5 Department of Molecular Biology, Regional Laboratory and Central Blood Bank, Abha, SAU; 6 Faculty of Pharmacy, Jazan University, Jazan, SAU; 7 Department of Pharmacy, Security Forces Hospital, Riyadh, SAU; 8 Department of Pharmacy, King Abdulaziz Specialist Hospital, Taif, SAU

**Keywords:** sars-cov-2, coronavirus disease 2019, newborns, infants, pregnant, sars-cov-2 infection, covid-19

## Abstract

Coronavirus disease 2019 (COVID-19) is a worldwide health problem, particularly for pregnant women. This review assesses the effects of COVID-19 on pregnant women and their infants. A systematic search was performed of studies published on PubMed, Web of Science, Google Scholar, and Embase from January 2020 to January 2021, without restriction by language. This review included 27 studies (22 from China, one from the United States, one from Honduras, one from Italy, one from Iran, and one from Spain), which cumulatively evaluated 386 pregnant women with clinically confirmed COVID-19 and their 334 newborns. Of the 386 pregnant women, 356 had already delivered their infants, four had medical abortions at the time of research, 28 were still pregnant, and two died from COVID-19 before they were able to give birth. Cesarean sections were performed on 71% of pregnant women with COVID-19 to give birth. Fever and cough were common symptoms among women. Premature rupture of membranes, distress, and preterm birth were pregnancy complications. Low birth weight and a short gestational age were common outcomes for newborns. The common laboratory findings among pregnant women were lymphopenia, leukocytosis, and elevated levels of C-reactive protein. Chest computed tomography revealed abnormal viral lung changes in 73.3% of women. Eleven infants tested positive for severe acute respiratory syndrome coronavirus 2 (SARS-CoV-2) infection. There was no evidence of vertical transmission. Most infants were observed to have lymphopenia and thrombocytopenia. The clinical features of pregnant women were found to be similar to those of generally infected patients. There is evidence of adverse pregnancy and neonatal outcomes caused by COVID-19.

## Introduction and background

The coronavirus disease 2019 (COVID-19) pandemic started in December 2019 in the Wuhan region of China. The causative agent of COVID-19 is severe acute respiratory syndrome coronavirus 2 (SARS-CoV-2). By November 2022, the virus had spread worldwide, with more than 600 million confirmed cases and more than 6 million deaths due to its high infection transmission rate [1,2]. Coronaviridae is a family of SARS-CoV-2 and is described as positive single-stranded RNA [3]. Regarding transmission, SARS-CoV-2 is transmitted more than other coronaviruses. COVID-19 is a systemic disease and can affect all humans [4].

Many studies have been conducted to define the clinical pictures and outcomes of the disease. However, a small number of studies have highlighted the impact of COVID-19 on pregnant women and their newborns. Pregnant women experience a variety of physiological and immunological changes, including elevation of the diaphragm, increased oxygen consumption, and edema of the respiratory tract mucosa, which can lead to hypoxia intolerance [5]. Pregnant women infected with COVID-19 are more susceptible to severe pneumonia and other symptoms due to immunosuppression [5].

Some data suggest that newborns can become infected with SARS-CoV-2 [6]. However, the correlation between COVID-19 infection during pregnancy and related outcomes is ambiguous. Therefore, we conducted this systematic review to assess the consequences of SARS-CoV-2 infection in pregnant women and their babies.

## Review

Methodology

Study Strategy and Selection Criteria

The study used the Preferred Reporting Items for Systematic Reviews and Meta-Analyses (PRISMA) guidelines [7].

Research Question

What are the consequences of SARS-CoV-2 infection in pregnant women and their newborns?

Search Strategy and Data Sources

We included studies published between January 2020 and January 2021 in PubMed, Web of Science, Google Scholar, and Embase, using the following keywords: "COVID-19" or "SARS-CoV-2" or "2019nCoV" or "coronavirus" and "pregnancy" or "pregnant" or "newborn" or "infant" or "child" or "neonatal," without any language restrictions. The papers were processed with EndNote® software (Clarivate, London, UK) to eliminate duplicates. Primary articles that evaluated the characteristics of COVID-19 during pregnancy were selected, and their titles and abstracts were screened for potential eligibility. Then, these studies were classified as relevant or irrelevant. The full texts of the relevant identified studies were read to determine final eligibility.

Inclusion Criteria

We included studies that involved pregnant women with COVID-19 and had data from positive COVID-19 cases performed using real-time reverse transcription polymerase chain reaction (RT-PCR), with the availability of descriptions of clinical characteristics, chest radiograph images, laboratory findings, treatment at admission, and neonatal outcomes.

Exclusion Criteria

Studies that did not have complete findings, studies that did not have access to data, and systematic reviews (to avoid overlapping data) were all excluded.

Data Extraction

We extracted the authors’ names, study design, place, sample size, clinical findings for pregnant women, pregnancy outcomes in the event of delivery, and primary limitations.

Outcome of Interest

The clinical features of pregnancy, maternal comorbidities, obstetric difficulties, clinical findings, delivery procedures, medicines given to infected pregnant mothers, mothers' deaths, the chance of vertical transmission of infection, and neonatal outcomes were accessed.

Statistical Analysis

Information on outcomes of interest was gathered and the data were entered into an Excel® spreadsheet (Microsoft Corporation, Redmond, WA) for evaluation. Categorical variables were expressed as numbers and percentages.

Results

**Figure 1:**
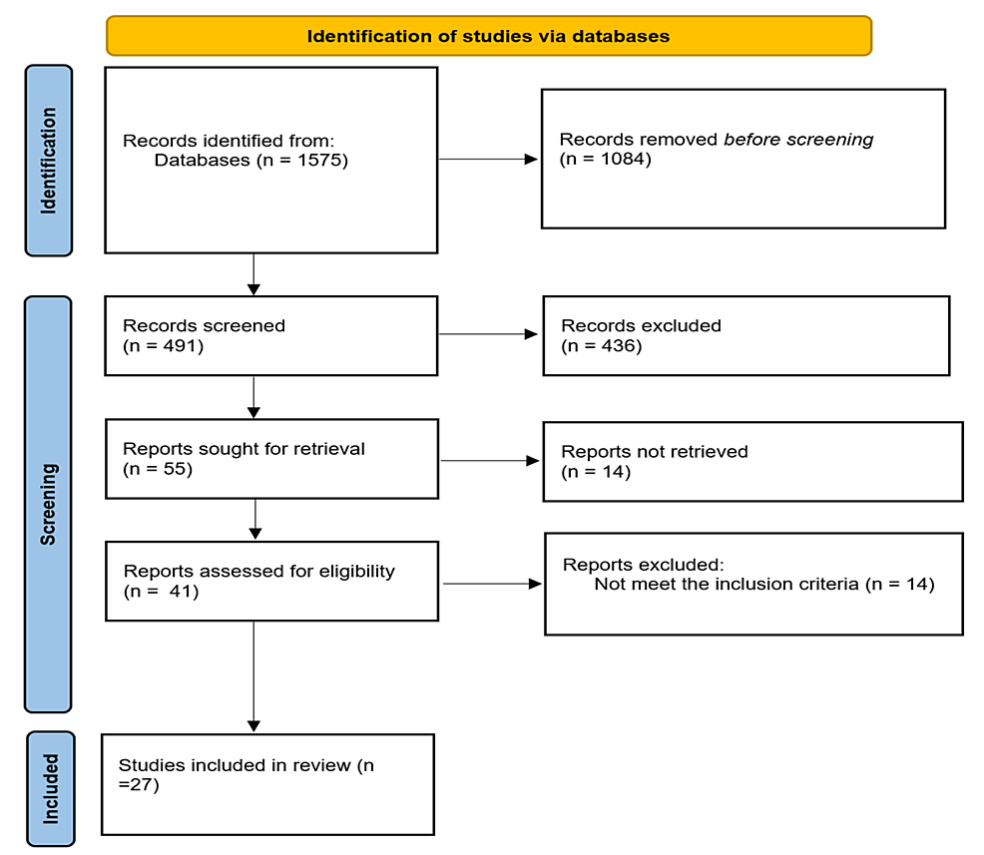
Study selection

In this systematic review, 1,575 studies were recognized in four databases. Of these, 1,084 were excluded due to duplicates. Of the 491 remaining studies, 450 were excluded because they did not evaluate pregnant women with COVID-19. Forty-one abstracts were selected, 14 of which were excluded because they did not meet the inclusion criteria. Finally, 27 studies were eligible for inclusion in the study. The search process was summarized in a flow diagram (Figure 1).

Among the 27 studies, 17 were retrospective, five were case series, four were case reports, and one was a cohort study. Twenty-two studies were carried out in China, one in the United States, one in Honduras, one in Italy, one in Iran, and one in Spain. This study involved 386 pregnant women who had positive results for SARS-CoV-2 using real-time RT-PCR to confirm COVID-19 infection. Most of them were evaluated for clinical characteristics and other complications during pregnancy and postpartum. Of pregnant women investigated, 67% were evaluated using additional laboratory tests, such as complete blood count, kidney and liver function tests, as well as C-reactive protein (CRP) tests. Of the 386 pregnant women with COVID-19, 356 had a successful delivery, four had medical abortions, 28 were still pregnant at the time of research, and two died from COVID-19 before they were able to deliver. The pregnancies produced 360 newborns (352 singletons and four sets of twins). Of the newborns, 71% were delivered by cesarean section due to pregnancy complications (i.e., fetal distress and placental disorders). Cesarean sections were also performed to mitigate the possibility of vertical transmission of SARS-CoV-2 to neonates (Table 1).

**Table 1:** Studies according to study design, place, sample size, clinical findings of pregnant women and neonate outcomes, study quality, and main limitations

Study	Study design	Country, city	Number of women	Findings of pregnant women with COVID-19	Neonatal outcomes	Main limitations
Zeng et al. (2020) [8]	Cohort study	China, Wuhan	33	33 patients were assessed for clinical features, and 3 cases had complications. 26 delivered by C-section and 7 delivered vaginally	33 neonates were assessed. 30 neonates tested negative for SARS-CoV-2 and 3 neonates tested positive for SARS-CoV-2	Single place. The study included only pregnant women in 3rd trimester. Pregnant patients were not assessed for lab findings
Chen et al. (2020) [9]	Retrospective	China, Wuhan	17	17 patients were assessed, and 8 cases had maternal comorbidities. All 17 were delivered by C-section	17 neonates were assessed. All 17 neonates tested negative for SARS-CoV-2	Single place. The study included only pregnant women in 3rd trimester
Zhang et al. (2020) [10]	Retrospective	China, Wuhan	16	16 patients were assessed for clinical features after treatment, and 14 cases had maternal comorbidities. All 16 were delivered by C-section. 15 were normal cases and 1 case was severe. None developed severe pneumonia	10 neonates were assessed for clinical findings. 10 neonates tested negative for SARS-CoV-2	Single place. The study included only pregnant women in 3rd trimester. Pregnant patients were not assessed for lab findings
Chen et al. (2020) [11]	Retrospective	China, Wuhan	9	9 patients were assessed. 7 cases had maternal comorbidities. All 9 were delivered by C-section. None developed severe pneumonia	9 neonates were assessed. 8 neonates tested negative for SARS-CoV-2 and 1 neonate tested positive 36 h after birth	Single place. The study included only pregnant women in 3rd trimester
Liu et al. (2020) [12]	Retrospective	China, nine cities	13	13 pregnant women diagnosed with COVID-19, 10 delivered babies. 3 were in the second trimester. Tests were not assessed. 5 cases had maternal comorbidities. All 10 were delivered by C-section	9 neonates tested negative for SARS-CoV-2. 1 neonate died. Neonates were not assessed for further lab tests	Neonates were not assessed for clinical features. Mothers and neonates were not assessed for lab findings
Zhu et al. (2020) [13]	Retrospective	China, Wuhan	9	9 patients were assessed for clinical features. 8 cases had maternal comorbidities. 2 were delivered vaginally and 7 delivered by C-section	10 neonates (including 2 twins) tested negative for SARS-CoV-2. 10 neonates were assessed	The study included only pregnant women in 3rd trimester. Pregnant patients were not assessed for lab findings
Liu et al. (2020) [14]	Retrospective	China, Wuhan & Shanghai	16	16 patients were assessed. 16 confirmed cases with SARS-CoV-2. 8 cases had maternal comorbidities. 16 delivered by C-section	All 16 neonates tested negative for SARS-CoV-2	Single place. Neonates were not assessed for clinical findings
Yu et al. (2020) [15]	Retrospective	China, Wuhan	7	7 patients were assessed. 5 cases had maternal comorbidities. 7 delivered by C-section	2 neonates tested negative and 1 was positive for SARS-CoV-2. No evidence of vertical transmission	Single place. The study included only pregnant women in 3rd trimester. Neonates were not assessed for lab findings
Chen et al. (2020) [16]	Case series	China, Wuhan	4	4 patients were assessed. 2 cases had maternal comorbidities. 1 delivered vaginally and 3 delivered by C-section	4 neonates were assessed. All 4 neonates tested negative for SARS-CoV-2	Single place. The study included only pregnant women in 3rd trimester
Wang et al. (2020) [17]	Case report	China, Suzhou	1	1 patient was assessed. Patient delivered by C-section	1 neonate tested negative for SARS-CoV-2. No further tests were assessed	Single place. The study included only pregnant women in 3rd trimester
Liu et al. (2020) [18]	Retrospective	China, Wuhan	15	15 patients were assessed. 2 cases had maternal comorbidities. 4 were still pregnant (3 in the 2nd trimester). 10 delivered by C-section and 1 delivered vaginally	All 11 neonates tested negative for SARS-CoV-2	Single place. Neonates were not assessed for lab findings
Fan et al. (2021) [19]	Case series	China, Wuhan	2	2 patients were assessed. Both were delivered by C-section	2 neonates tested negative for SARS-CoV-2	Single place. The study included only pregnant women in 3rd trimester
Chen et al. (2020) [20]	Retrospective	China, Wuhan	3	3 patients were assessed. 2 confirmed cases and 1 clinically diagnosed. 3 cases had maternal comorbidities. All 3 delivered by C-section	3 neonates tested negative for SARS-CoV-2	Single place. The study included only pregnant women in 3rd trimester. Neonates were not assessed for lab findings
Chen et al. (2020) [21]	Retrospective	China, Wuhan	5	5 patients were assessed. 3 cases had maternal comorbidities. 3 delivered vaginally and 2 delivered by C-section	5 neonates tested negative for SARS-CoV-2	Single place. The study included only pregnant women in 3rd trimester
Breslin et al. (2020) [22]	Case series	United States. New York	7	7 patients were assessed. 3 cases had maternal comorbidities. 2 delivered vaginally and 5 were still pregnant	2 neonates tested negative for SARS-CoV-2	Single place. 2 neonates were not assessed for clinical features and lab findings
Zambrano et al. (2020) [23]	Case report	Honduras, Tegucigalpa	1	1 patient was assessed. The case had maternal comorbidities. The patient delivered vaginally	1 neonate tested negative for SARS-CoV-2	Single place. The mother and neonate were not assessed for lab findings
Wang et al. (2020) [24]	Case report	China, Wuhan	1	1 patient was assessed. The patient was delivered by C-section. The case had maternal comorbidities	1 neonate tested positive for SARS-CoV-2 36 hours after birth	Single place. The study included only pregnant women in 3rd trimester
Liu et al. (2020) [25]	Case series	China, Wuhan	3	3 patients were assessed. 3 cases had maternal comorbidities. 1 delivered vaginally and 2 delivered by C-section	3 neonates tested negative for SARS-CoV-2	Single place. The study included only pregnant women in 3rd trimester
Li et al. (2020) [26]	Retrospective	China, Wuhan	16	16 patients were assessed. 13 cases had maternal comorbidities. 2 delivered vaginally and 14 were delivered by C-section	17 neonates (including 2 twins) tested negative for SARS-CoV-2	Single place. The study included only pregnant women in 3rd trimester
Liao et al. (2020) [27]	Case report	China, Chongqing	1	1 patient was assessed. Patient delivered by C-section	1 neonate was assessed. Neonate tested negative for SARS-CoV-2	Single place. The study included only pregnant women in 3rd trimester. Neonate was not assessed for clinical features and lab findings
Ferrazzi et al. (2020) [28]	Retrospective	Italy, Milan, Mangiagalli and Sacco	42	42 patients were assessed. 6 cases had maternal comorbidities. 24 delivered vaginally and 18 delivered by C-section	42 neonates were assessed. 38 tested negative for SARS-CoV-2 and 2 tested positive for SARS-CoV-2 due to direct contact with infected mothers. 1 tested positive after vaginal delivery	The study included only pregnant women in 3rd trimester. Neonates were not assessed for clinical findings
Xu et al. (2020) [29]	Retrospective	China, Wuhan	28	28 patients were assessed. 8 cases had maternal comorbidities. 17 delivered by C-section and 5 delivered vaginally. 4 had medical abortions. 2 were still pregnant	26 neonates tested negative for SARS-CoV-2. 23 neonates were assessed	Neonates were not assessed for lab findings
Xu et al. (2020) [30]	Retrospective	China, Wuhan	5	5 patients were assessed. 4 cases had maternal comorbidities. 4 delivered by C-section and 1 delivered vaginally	5 neonates were assessed. 5 tested negative for SARS-CoV-2	Single place. The study included only pregnant women in 3rd trimester. Neonates were not assessed for lab findings
Hantoushzadeh et al. (2020) [31]	Case series	Iran, Tehran	9	9 patients were assessed. 7 patients died. 6 cases had maternal comorbidities. 7 were delivered by C-section and 2 died undelivered	10 neonates (including twins) were assessed. 6 tested negative for SARS-CoV-2 and 4 died	Neonates were not assessed for clinical features
Cao et al. (2020) [32]	Retrospective	China, Wuhan	10	10 patients were assessed. 9 cases had maternal comorbidities. 2 were delivered vaginally and 8 delivered by C-section. 6 cases had mycoplasma pneumonia	11 neonates (including twins) were assessed. 5 tested negative for SARS-CoV-2	Single place. The study only included pregnant women in the 3rd trimester of pregnancy. Neonates were not assessed for lab findings
Yin et al. (2020) [33]	Retrospective	China, Wuhan	31	31 patients were assessed. 14 were still pregnant. 17 gave birth. 13 delivered by C-section and 4 delivered vaginally. 3 cases had maternal comorbidities	17 neonates tested negative for SARS-CoV-2	Single place
Martínez-Perez et al. (2020) [34]	Retrospective	Spain, Madrid	82	82 patients were assessed. 41 were delivered by C-section and 41 were delivered vaginally. 27 cases had maternal comorbidities. 4 cases had severe COVID-19 symptoms	82 neonates were assessed. 67 neonates tested negative for SRAR-CoV-2 and 2 neonates tested positive for SARS-CoV-2 (both had direct contact with their mothers). 3 suspected neonates with COVID-19	Neonates were not assessed for lab findings. Lack of sufficient information on newborns to determine vertical transmission

Our results revealed that 91%, 7%, and 2% of pregnant women were in the third, second, and first trimesters, respectively. The pregnant patients ranged in age from 19 to 48 years old. Fever was recorded in 44.7% of all cases, followed by cough (31.2%), postpartum fever (14.8%), breathing difficulties (13.4%), myalgia (7.8%), fatigue (7.2%), diarrhea (3.9%), chest distress (3.2%), sore throat (2.6%), and headache (1.9%) (Table 2).

**Table 2:** Maternal characteristics, clinical characteristics, comorbidities, complications, co-infections, abnormal findings, and maternal outcomes experienced by women with COVID-19 admitted for delivery PROM: premature rupture of membranes; CRP: C-reactive protein; ALT: alanine transaminase; AST: aspartate transaminase; BUN: blood urea nitrogen; N: number.

Maternal characteristics	n/N	%
Infected maternal	386	100%
Age, year (min-max)	(19-48)	33 (median)
Advanced maternal age > 35	14/304	4.6%
Clinical features		
Fever	136/304	44.7%
Cough	95/304	31.2%
Postpartum fever	45/304	14.8%
Breathing difficulty	41/304	13.4%
Myalgia	24/304	7.8%
Fatigue	22/304	7.2%
Diarrhea	12/ 304	3.9%
Chest distress	10/304	3.2%
Sore throat	8/ 304	2.6%
Headache	6/304	1.9%
Malaise	3/304	0.9%
Poor appetite	2/304	0.6%
Maternal comorbidities		
Diabetes mellitus/gestational diabetes	32/386	8.3%
Hypertension/gestational hypertension	14/386	3.6%
Hypothyroidism	14/386	3.6%
Anemia	10/386	2.5%
Asthma	8/386	2.1%
Scarred uterus	7/386	1.8%
Hepatitis B	5/386	1.2%
B-lynch suture	2/386	0.5%
Obesity	2/386	0.5%
Polycystic ovary syndrome	2/386	0.5%
Cardiovascular disease	2/386	0.5%
Multiple organs dysfunction syndrome	1/386	0.2%
Underweight	1/386	0.2%
Hepatitis C	1/386	0.2%
Complications		
PROM	23/386	5.9%
Fetal distress	19/386	4.9%
Abdominal pain	5/386	1.2%
Cholecystitis	4/386	1.0%
Placenta previa	4/386	1.0%
Vaginal bleeding	4/386	1.0%
Anorexia	2/386	0.5%
Acute renal failure	1/386	0.2%
Acute hepatic failure	1/386	0.2%
Placenta abruption	1/386	0.2%
Skin rash	1/386	0.2%
Co-infection		
Mycoplasma	6/50	12%
Influenza	2/50	4%
Legionella pneumophila	1/50	2%
Maternal outcomes		
Cesarean section	255/356	71.6%
Operative vaginal delivery	97/356	27.2%
Medical abortion	4/356	1.1%
Still pregnant	28/386	7.2%
Died undelivered	2/386	0.50%
Critical cases	14/386	3.6%
Total maternal deaths	7/386	1.8%
Abnormal laboratory findings and CT imaging		
Leukocytosis	63/197	31.9%
Leucopenia	15/197	7.6%
Lymphocytopenia	92/261	35.2%
Thrombocytopenia	12/63	19.0%
High level of CRP	104/172	60.4%
High level of AST-ALT	27/209	12.9%
High level of BUN-creatinine	1/65	1.5%
Chest CT evidence of pneumonia	283/386	73.3%
Type of intrauterine results		
Placenta (positive results)	0/2	0.0%
Cord blood (positive results)	0/10	0.0%
Amniotic fluid (positive results)	0/40	0.0%
Breast milk (positive results)	0/40	0.0%

Medical history revealed that 101 pregnant women had chronic diseases prior to COVID-19 infection, the most significant of which were diabetes mellitus (8.3%), hypertension (3.6%), hypothyroidism (3.6%), anemia (2.5%), asthma (2.1%), scarred uterus (1.8%), hepatitis B (1.2%), B-lynch suture (1%), obesity (1%), polycystic ovary syndrome (1%), cardiovascular disease (1%), multiple organ dysfunction syndrome (0.2%), hepatitis B (0.2%), and underweight (0.2%) (Table 2).

The pregnancy complications that developed included premature rupture of membranes (PROM, 5.9%), fetal distress (4.9%), abdominal pain (1.2%), cholecystitis (1%), placenta previa (1%), vaginal bleeding (1%), anorexia (0.5%), acute renal failure (0.2%), acute liver failure (0.2%) placenta abruption (0.2%), and skin rash (0.2%). Only 3.6% were critical and had to be admitted to the ICU. Of these, 1.8% resulted in death (Table 2).

Laboratory findings of pregnant women showed that lymphocyte counts were normal in 168 cases (64%), lower than normal in 92 cases (35.2%), and higher than normal in one case (0.3%). Platelet counts were normal in 51 cases (81%) and low in 12 cases (19%); no increase in platelet count was observed. High levels of CRP were reported in 104 patients (60%), while 68 patients (40%) were within the normal range. Elevated liver enzymes (alanine transaminase (ALT) and aspartate transaminase (AST)) were reported in 27 patients (13%), while 182 patients (87%) were within the normal range. Renal function tests included blood urea nitrogen (BUN) and serum creatinine (Cr); these were at normal, decreased, and increased concentrations in 61 cases (95%), two cases (3%), and one case (2%), respectively (Table 2).

Co-infection with other pathogens was observed in four studies. Six pregnant patients tested positive for mycoplasma pneumonia, two for influenza, and one for *Legionella pneumophila*. Of pregnant women examined with COVID-19, 356 gave birth, producing 360 newborns (352 singletons and four sets of twins). Of these 356 women, 255 (71%) delivered their infants by cesarean section, 97 (27.2%) had natural vaginal deliveries, and four had medical abortions. There was no evidence of vertical transmission; umbilical cord blood, amniotic fluid, breast milk samples, and the placenta were negative using real-time RT-PCR (Table 2).

Antibiotic therapy was administered to 94 patients (54%) to treat infections. Of these, 20 (11.5%) received cephalosporins, four (2.3%) received quinolones, two (1.1%) received a combination of azithromycin and ceftazidime, one (0.5%) received a combination of cefoperazone sodium and sodium sulbactam, and one (0.5%) received azithromycin. Antiviral was administered in combination with antibiotics in 81 women (46%). Specifically, 23 (13.9%) received oseltamivir, 20 (11.5%) received ribavirin, 11 (6.3%) received interferon, nine (5.2%) received ganciclovir, eight (4.6%) received Arbidol, seven (4%) received lopinavir, two (1.1%) received abedore hydrochloride, and one patient (0.5%) received umifenovir. Other therapies, such as steroids (mainly methylprednisolone), were administered to nine (5.7%) of the patients. Two studies reported using hydroxychloroquine in eight cases (4.6%), and a study in China administered traditional Chinese medicine to two patients (1.1%). A total of 128 patients (50%) received oxygen support through a nasal cannula. Two studies administered gamma globulin intravenously to six patients (3.4%). Only one patient with multiple organ system dysfunctions was treated with extracorporeal membrane oxygenation (ECOM). Most studies did not describe the drug dose, route of administration, and duration or timing of treatment (Table 3).

**Table 3:** Treatments given to mothers infected with SARS-CoV-2

Treatment	n/N	%
Antiviral therapy	81/173	46·8%
Oseltamivir	23/173	13·9%
Ribavirin	20/173	11·5%
Interferon	11/173	6·3%
Ganciclovir	9/173	5·2%
Arbidol	8/173	4·6%
Lopinavir/ritonavir	7/173	4·0%
Abedore hydrochloride	2/173	1·1%
Umifenovir	1/173	0·5%
Antibiotic therapy	94/173	54·3%
Cephalosporins	20/173	11·5%
Quinolones	4/173	2·3%
Azithromycin + ceftazidime	2/173	1·1%
Cefoperazone sodium + sulbactam sodium	1/173	0·5%
Azithromycin	1/173	0·5%
Moxifloxacin + glucocorticoid	1/173	0·5%
Use of corticosteroid	9/173	5·7%
Methylprednisolone	9/173	5·7%
Traditional Chinese medicine	2/173	1·1%
Lianhua Qingwen capsule & Jinyebaidu granules	2/173	1·1%
Other therapies	61/173	35·2%
Oxygen support (nasal cannula)	128/255	50·1%
Hydroxychloroquine	8/173	4·6%
Gamma globulin	6/173	3·4%
Extracorporeal membrane oxygenation (ECOM)	1/173	0·5%

The relevant data on babies born to COVID-19-positive mothers are included in Table 4. Of the articles included in the review, 24 studies reported on 334 newborns. The weight of newborns ranged from 910 to 4,750 g. Forty-two (12.5%) newborn babies had a birth weight of less than 2,500 g. Sixty-four (19.1%) were small for gestational age (SGA). Ten (2.9%) newborns had an abnormal APGAR (appearance, pulse, grimace, activity, and respiration) score. Fifty-two babies (15.5%) were admitted to the neonatal intensive care unit (NICU); six of them died (2.3%). Throat swabs using real-time RT-PCR were used to test 301 neonates for infection with SARS-CoV-2. Of these 301 babies, 11 tested positive for SARS-CoV-2 between six and 36 hours after birth. Ten of the positive infants were delivered by cesarean section and one was delivered vaginally. None of them developed severe complications (Table 4).

**Table 4:** Neonatal outcomes NICU: neonatal intensive care unit; RT-PCR: reverse transcription polymerase chain reaction; DIC: disseminated intravascular coagulation; RDS: respiratory distress syndrome; APGAR: appearance, pulse, grimace, activity, and respiration.

Neonatal outcomes	n/N	%
Assessed infants		
Small for gestational age	64/334	19·1%
Low body weight	42/334	12·5%
NICU admission	52/334	15·5%
SARS-CoV-2 infected	11/334	3·2%
Abnormal APGAR	10/334	2·9%
Death	6/334	1·7%
Clinical findings		
CT pneumonia	17/76	22·3%
Lymphocytopenia	15/35	42·8%
Positive for RT-PCR	9/219	4·1%
Leukocytosis	5/32	15·6%
Thrombocytopenia	4/24	16·6%
Leukopenia	3/32	9·3%
Clinical features		
Short of breath	12/269	4·4%
Asphyxia	9/269	3·3%
Pneumonia	9/269	3·3%
Cyanosis	6/269	2·2%
Feeding intolerance	5/269	1·8%
Fever	5/269	1·8%
RDS	4/269	1·4%
Vomiting	3/269	0·7%
Complications		
Secondary bacterial infection	5/187	2·6%
Skin rash & edema	4/187	2·1%
Gastric bleeding	2/187	1·1%
Multiple organ failure & DIC	1/187	1·1%
Mild bloating	1/187	1·1%
Treatment		
Antibiotic therapy	9/42	21·4%
Mechanical ventilation	2/42	4·7%
Antiviral therapy	0/42	0·0%
Other therapies		
Platelet transfusion	2/10	20%
Red blood cell transfusion	1/10	10%
Plasma transfusion	1/10	10%
Gamma globulin transfusion	1/10	10%

Laboratory findings and chest CT scans were used to test 35 neonates, which revealed that five (15.6%) had leukocytosis, three (9.3%) had leukopenia, and 75.8% were within the normal range. Thirty-five newborns were tested for lymphocyte count; 15 (42.8%) had lymphopenia and 20 (57.1%) had a normal lymphocyte count. Platelet count assessments were performed on 24 neonates; four (16.6%) had thrombocytopenia, and the rest were within the normal range. Finally, 76 neonates underwent chest CT scans; 17 (22.3%) showed abnormalities, the most prevalent of which were opacity of "ground glass," patchy "shadows," and consolidations (Table 4).

Neonatal symptoms were reported and the clinical characteristics of 178 neonates were assessed. Of these, 12 (4.4%) had shortness of breath, nine (3.3%) had asphyxia, nine (3.3%) had pneumonia, six (2.2%) had cyanosis, five (1.8%) had a fever, five (1.8%) had food intolerances, four (1.4%) had respiratory distress syndrome (RDS), and three (1%) experienced vomiting. Furthermore, 12 of the 187 neonates experienced complications, such as sepsis (n = 5, 2.6%), skin rashes and edema (n = 4, 2.1%), gastric bleeding (n = 2, 1.1%), and refractory shock (n = 1, 0.6%), which resulted in multiple organ failure and disseminated intravascular coagulation (DIC). The newborn with DIC was treated with platelet transfusion, suspended red blood cells, and plasma; he died on the ninth day. Another neonate with gastric bleeding was treated with platelet transfusion, suspended red blood cells, plasma, and gamma globulin; he recovered in 15 days. Nine newborns received antibiotics and two were supported with noninvasive mechanical ventilation. None of the newborns were given antiviral (Table 4).

Discussion

Across the study, 386 pregnant women with COVID-19 were evaluated. Of these, almost all were in their third trimester (91%), which explains the birth and inclusion of 334 newborns in this review. Due to PROM and fetal distress, most women underwent cesarean sections to avoid vertical transmission and reduce adverse perinatal and neonatal outcomes [9,35]. Elective cesarean sections were also administered to minimize the respiratory distress of mothers [6,9]. In this study, most pregnant women were asymptomatic. Symptomatic pregnant women developed mild to moderate symptoms, most commonly fever, nonproductive cough, and postpartum fever, consistent with the results of a previous study [35]. In our study, pregnancies in the early gestational stages were discharged without severe complications. However, due to a lack of data on their perinatal outcomes, it was not possible to interpret neonatal outcomes when the infection is acquired early in pregnancy.

A total of 26% of women had chronic diseases; the top five identified comorbidities were diabetes mellitus, hypertensive disorders, thyroid disease, placental disorders, and anemia. Among the obstetric complications were PROM, fetal distress, preterm labor, acute cholecystitis, placenta previa, and vaginal bleeding in the third trimester, which are consistent with previous studies [35]. The presence of comorbidities increased the risk of poor outcomes and maternal death. Therefore, a proper patient classification must be performed carefully, documenting their medical history. This will help identify which pregnant patients are at high risk for poor outcomes related to COVID-19 [12].

The most significant laboratory findings among pregnant women were lymphocytopenia, leukocytosis, and elevated liver enzymes. Consolidation lesions and patchy ground glass shadows were the most prevalent abnormalities identified on chest CT scans. Pre- or postpartum maternal death due to infection is an area of concern. Seven mothers with COVID-19 were reported to have died in Iran [31]. Three, two, and two of the deaths were related to advanced maternal age, comorbidities, and acute RDS, respectively; two occurred during the second and third trimesters. This suggests that advanced maternal age and comorbidities with COVID-19 could increase the mortality rate in pregnant patients with COVID-19 [31]. However, more data from other countries on pregnant with COVID-19 are needed to confirm maternal mortality from COVID-19.

Pregnant women received the standard treatment used to lessen the severity of their infection. Ninety-four women received individual antibiotics or antibiotics in combination with steroids, mainly methylprednisolone, to prevent bacterial superinfection. A total of 128 patients received oxygen support through a nasal cannula. Antiviral therapies commonly administered to infected pregnant women, such as oseltamivir, ribavirin, interferon, ganciclovir, arbidol, and lopinavir, were also administered. Other studies have suggested the use of hydroxychloroquine during pregnancy [36]. In the current study, traditional Chinese medicine was used in two cases in China. Another patient with multiple organ system dysfunctions was treated with ECOM. It is important to note that physicians must exercise caution when prescribing any antiviral therapy to infected pregnant women.

Of the 334 newborns examined, 42 were born with low birth weight and 64 were preterm. Of all cases that presented evidence of pneumonia following a CT scan, 15 had lymphocytopenia. Commonly encountered neonatal complications, such as skin rash, edema, gastric bleeding, stillbirth, and neonatal death, were not found to be significantly related to SARS-CoV-2 infection. Six newborns found negative for SARS-CoV-2 died. These data demonstrate that maternal COVID-19 had significant adverse effects on newborns. As such, extra attention and care must be paid to newborns of mothers infected with COVID-19, in accordance with a previous study [13].

Eleven of the 290 neonates observed in the current review were clinically healthy and did not experience any complications; however, most of them had lymphopenia and thrombocytopenia. Three had evidence of pneumonia. Although three of the studies included in the review did not identify SARS-CoV-2 in the placenta, cord blood, amniotic fluid, or breast milk samples, it was not possible to determine whether transmission occurred vertically or through direct contact. Therefore, more studies are warranted in this regard.

Limitations and strengths

The first limitation was that most pregnant women were in the third trimester of their pregnancy. Therefore, any interpretations of maternal and neonatal outcomes for infections that were acquired early in pregnancy require further validation. The second limitation was that most of the studies were conducted in China, so the findings cannot be generalized to other populations. The strengths and findings of this review can be used to inform healthcare providers of the clinical and radiological characteristics, laboratory findings, and recommended treatment of pregnant women with COVID-19, as well as the neonatal outcomes of mothers with COVID-19.

## Conclusions

The clinical features of pregnant women with COVID-19 were similar to those of general COVID-19 patients. There was no evidence of intrauterine transmission of SARS-CoV-2 in the third trimester, as SARS-CoV-2 was not detected in the placenta, cord blood, amniotic fluid, or breast milk samples. The risks of adverse pregnancy and neonatal outcomes in mothers with COVID-19 were demonstrated. There was evidence of complications such as premature membrane rupture, fetal distress, and preterm delivery. Small size for gestational age, low birth weight, lymphopenia, and thrombocytopenia were common adverse neonatal outcomes. The primary laboratory findings among pregnant women were lymphopenia, leukocytosis, and elevated CRP concentrations. Additional research is necessary to evaluate the long-term impact of COVID-19 on pregnancy and newborn outcomes.
